# A review on recent advances in the electrochemical reduction of CO_2_ to CO with nano-electrocatalysts

**DOI:** 10.1039/d2ra03341k

**Published:** 2022-08-15

**Authors:** Kee Chun Poon, Wei Yang Wan, Haibin Su, Hirotaka Sato

**Affiliations:** School of Mechanical & Aerospace Engineering, Nanyang Technological University 50 Nanyang Avenue Singapore 639798 hirosato@ntu.edu.sg keechun.poon@ntu.edu.sg; Department of Chemistry, The Hong Kong University of Science and Technology Clear Water Bay Hong Kong China

## Abstract

The electrochemical reduction (ECR) of CO_2_ is a powerful strategy to reduce the world's carbon footprint by converting CO_2_ to useful products such as CH_3_OH and CO. Recent techno-economic analysis has found that for the electro-conversion of CO_2_ to be adapted for practical use, the main products formed from this reaction need to be low-order, such as CO. This review summarizes recent progress in the ECR of CO_2_ to CO on nano-electrocatalysts (noble, non-noble metals and carbon nanomaterials) and provides the limitations and challenges that each electrocatalyst faces. It discusses the mechanism behind the performance of the electrocatalysts and offers the potential future prospects of the ECR process.

## Abstract 2

Electrochemical reduction (ECR) of CO_2_ reduces world’s carbon footprint by converting CO_2_ to useful products such as CH_3_OH and CO. This review summarizes recent progress in the ECR of CO_2_ to CO on nano-electrocatalysts.

## Graphical abstract

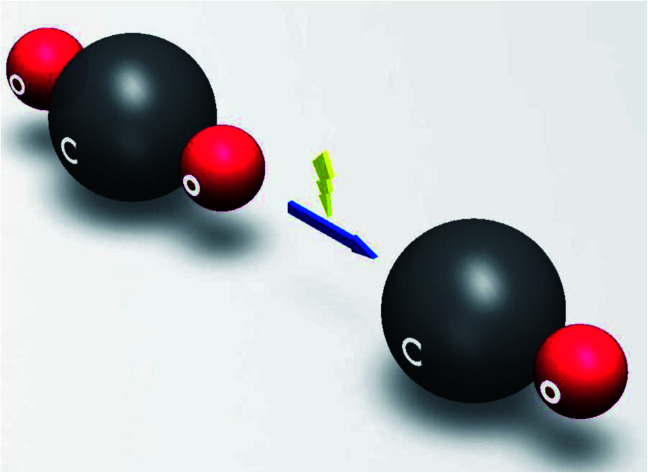

## 1. Introduction

The increase in energy demand over the past few centuries has led to a surge in the utilization of fossil fuel-based power sources such as petroleum, coal and natural gas.^1,2^ The result of this has been a surge in greenhouse emissions, causing severe environmental effects such as global warming, ocean acidification and desertification.^3–5^ The leading source of greenhouse emissions is carbon dioxide (CO_2_), which the Intergovernmental Panel on Climate Change (IPCC) has contributes up to 76% of all greenhouse gases.^6^ Therefore, there is an increasing need to solve the crisis of the extensive build-up of CO_2_ that the world has generated over the years. Additionally, carbon capture and utilization have been established by the work of Ghiat *et al.* to help with CO_2_ abatement.^69^ In another study done by Sun *et al.*, integrated CO_2_ capture and utilization (ICCU) reduced the path of CO_2_ usage and its conversion.^70^ Concepts and models have also been established by Garcia *et al.* for heterogeneous catalysts employed in CO_2_ conversion.^71^ Progress in electrochemical CO_2_ reduction on copper (Cu) in water-based electrolytes has also been established, together with a comprehensive study on the utilization and conversion of CO_2_.^72,73^

There are two common methods that researchers and industries have been focused on to tackle this problem. First would be a solution to the CO_2_ emission problem at the production level. This would involve a reduction in the dependence on fossil fuel and the search for alternative energy sources. These alternative energy sources include solar, wind, biomass, geothermal, hydro and biofuels, which are clean, renewable and most importantly do not produce any harmful CO_2_ by-products.^7–11^ However, despite the recent increase of these alternative sources from 5% in 2008 to almost 28% in 2020, their high production cost and limitations such as geographical constraints have limited the full switch to such alternatives.^12–14^ Furthermore, the effective use of such renewable energy relies heavily on energy conversion and storage technologies, which are limited by low energy density conversions and high production costs, which makes the large-scale production of such facilities difficult to accomplish.^15–17^ Therefore, an approach to overcome the CO_2_ production problem should be established.

The second approach would involve the carbon capture and utilization (CCU) method. The idea behind this is simple. The CO_2_ products are retrieved and converted into useful materials such as carbon monoxide (CO), formic acid (HCOOH), methanol (CH_3_OH), and methane (CH_4_).^18–21^ This strategy can effectively reduce the CO_2_ in the air, and also create fuel to produce useful and desirable industrial products. One example of this conversion is CO_2_ hydrogenation, where CO_2_ is converted to CH_3_OH.^22–24^ However, the main problem with combustion-based conversion is that the heat comes from fossil fuel, which substantially lowers the net amount of CO_2_ being converted.^25,26^

**Fig. 1:**
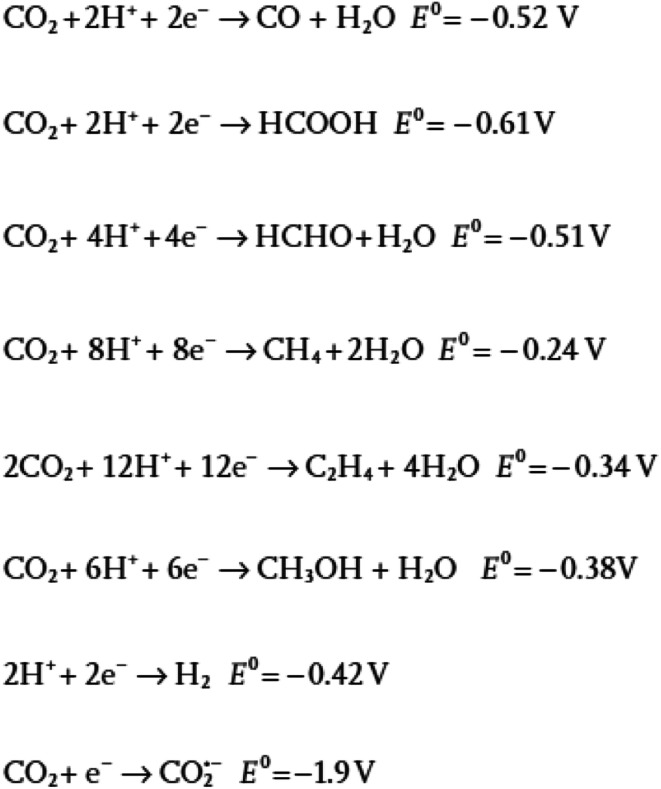
Thermodynamic potential difference of the products made by the electrochemical reduction of carbon dioxide (CO_2_). Reprinted with permission from ref. 31. Copyright 2016 Wiley.

Recently, electrochemical method has become an increasingly popular method in the conversion of CO_2_.^27–30^ This method, which is called the electrochemical reduction (ECR) of CO_2_, is particularly appealing for multiple reasons. Firstly, using the ECR technique, high selectivity of the desired products can be obtained, as seen in Fig. 1.^31^ The selectivity of the products can be actively controlled by simply adjusting the voltage (energy) supplied to the reaction. Furthermore, the energy supplied in the electrochemical reduction can be obtained from renewable sources, which means additional CO_2_ is not generated during this process.^32,33^ It should be noted that this process can be conducted at ambient pressure and temperature with little complications for its scaling up. Moreover, the reactor setup for the electroreduction process is modular, increasing the ease of its large-scale application.^34–36^ Lastly, the reactants consumed in the electroreduction process are simple such as water and CO_2_, while the other components that are not consumed such as the electrolyte and electrodes are reusable, which reduces both the cost and pollution.^34–36^

However, CO_2_ ECR still exhibits some drawbacks. Firstly and importantly, based on the recent techno-economic analysis conducted for industrial use (TEA survey), it was found that the ECR method is competitive for industrial application, where lowered order products such as carbon monoxide (CO) and formate ion (HCOO^−^) are preferred over high-order products such as CH_3_OH.^32,33^ The faradaic efficiency (FE) of CO, which corresponds to the high current density, is higher than that of the other intermediates. This is due to the high activation barrier requirement of the ECR process, which is related to the next point, *i.e.*, the structure of CO_2_. Due to the linear nature of the CO_2_ molecule, its conformation needs to be changed to the bent CO_2_˙^−^ to become activated towards the electroreduction process, as seen in Fig. 3.^31,37^ This requirement results in a high energy barrier for the formation of the CO_2_˙^−^ intermediate, which translates to a large overpotential requirement (the difference between the equilibrium and onset potential). Ultimately, this results in poor energy efficiency for the electroreduction process. Furthermore, the kinetics of the electroreduction reaction is slow with the limited mass transfer process of CO_2_, which results in sluggish reaction rates for the reduction of CO_2_, and thus a long processing time is needed.^38,39^ Another drawback is the CO_2_ electroreduction in aqueous solvents, where the competing reaction of the hydrogen evolution reaction (HER) decreases the overall production efficiency of the reaction.^40–42^ Also, the electrocatalyst used for the electroreduction process can be poisoned by the intermediates formed during the process, which can deactivate the catalyst and incur greater labour and replacement costs. Finally, mixed products can be formed during the ECR process, which adds another overhead on the production line due to the need for product separation.

## 2. Understanding the fundamentals and evaluation of electrochemical reduction of CO_2_ to CO

**Fig. 2:**
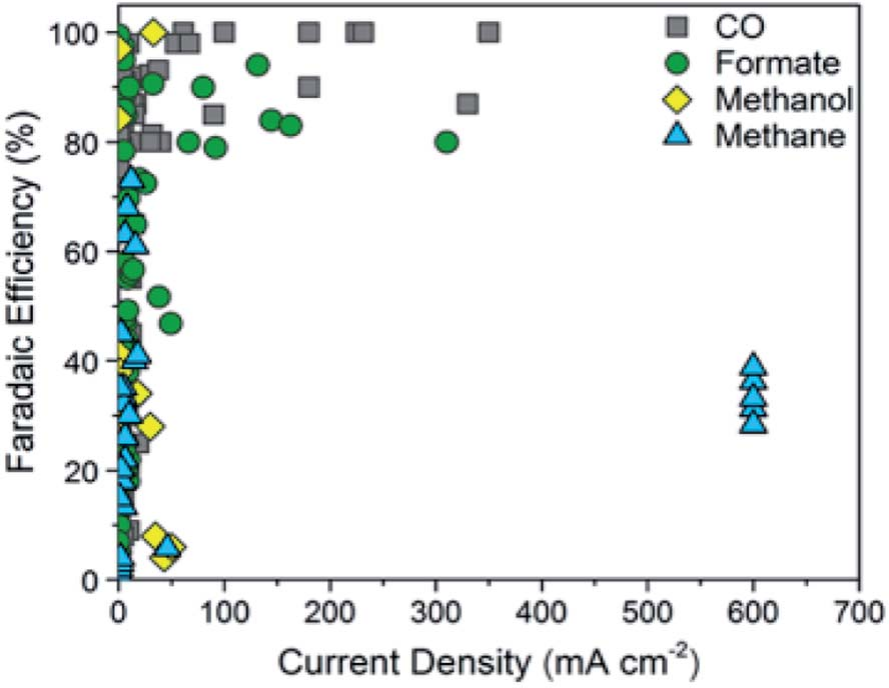
Faradaic efficiency *versus* current density for carbon monoxide. Reprinted with permission from ref. 32. Copyright 2018, ACS Publications.

The ECR of CO_2_ to CO is a multi-step reaction, which involves a two-electron transfer process, as seen in Fig. 2.^31^ Overall, for the ECR to CO, there are 3 main steps. Firstly, the adsorption of CO_2_ on the surface of the electrocatalyst. This is followed by the reduction of CO_2_ to form the CO_2_˙^−^ intermediate. The final step is the reduction (typically with another CO_2_ molecule) to produce CO.

To understand and evaluate the electrocatalyst performance towards ECR to CO, there are certain definitions and comparison methods that need to be clarified beforehand, as follows:

### 2.1 Onset potential and overpotential

The onset potential is the potential applied to the electrocatalyst (*vs.* the reference electrode) for CO to be detected. The overpotential is the difference between the onset potential and the equilibrium potential of the ECR to CO reaction. Therefore, a desirable electrocatalyst should require the greatest positive onset potential, while having the smallest possible overpotential (the best value is the same as that for equilibrium).

### 2.2 Current density

This is obtained by normalizing the current by either the surface area of the electrode or the electrocatalyst. This value indicates the amount of reaction occurring during the ERC to CO. It is also used in the calculation of the faradaic efficiency (FE), which is discussed in the following point. Also, this value is important especially from an industrial application point of view given that it will determine the production and operation cost of running the ECR setup.

### 2.3 Faradaic efficiency (FE)

This is defined as the percentage of CO formed by the amount of electrons used in the ECR. The FE is calculated using eqn (1), as follows:1
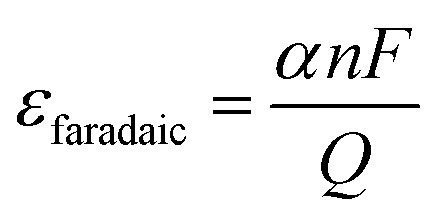
where *α* is the number of electrons transferred in the reaction, *n* is the number of moles of CO formed during the ECR, *F* is Faraday's constant (96 485 C mol^−1^) and *Q* is the amount of charge passed during the ECR. The FE enables the understanding of the percentage of CO produced in the ECR process.

## 3. Electrocatalysts

Recently, a variety of electrocatalysts has been used for the ECR to CO process. They can be classified into 2 main categories, *i.e.*, metal and non-metal electrocatalysts, each with its own advantages and disadvantages. The metal electrocatalysts can be further split into 2 sub-categories, *i.e.*, noble metals and non-noble metals. This review explores the recent advances in each of these categories together with its shortcomings and potential applications.

### 3.1 Noble metals

In the case of noble metals, in the past decade, gold (Au), silver (Ag) and palladium (Pd) have been the three most commonly explored metals for the electro-conversion of CO_2_ to CO. This is due to several factors, which will be explained in detail later.

#### 3.1.1 Gold (Au)

**Fig. 3:**
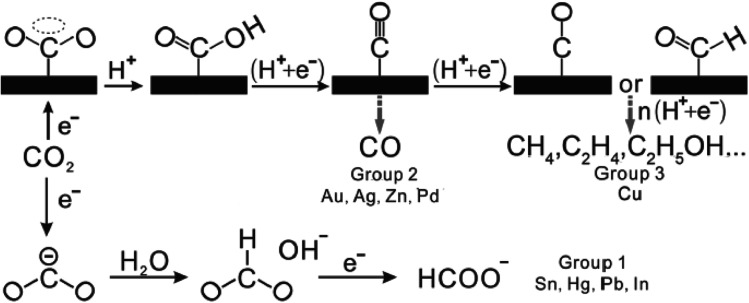
Reaction pathway for the electrochemical reduction of CO_2_. Reprinted with permission from ref. 31. Copyright 2016, Wiley.

Due to its high conversion rate of CO_2_ to CO, Au has been studied extensively as the ideal catalyst for the ECR process. This is further supported by previous computational studies, where Au was shown to be located at the top of the volcano plot, as seen in Fig. 3.^43^ This means is that Au has the optimal binding energy for the key intermediate of COOH, which is essential in the ECR process to CO. This also translates into an overall lower overpotential for the ECR process and highest catalytic activity and FE among the catalysts. In comparison to other electrocatalysts, Au has the highest binding energy with respect to the COOH intermediates. However, the rarity and high cost of Au severely limit its actual application. Accordingly, many recent studies have been conducted to address this issue by optimizing and further improving the cost efficiency of Au by methods such as reducing its particle size and changing its morphology. This is further summarized below.

**Fig. 4:**
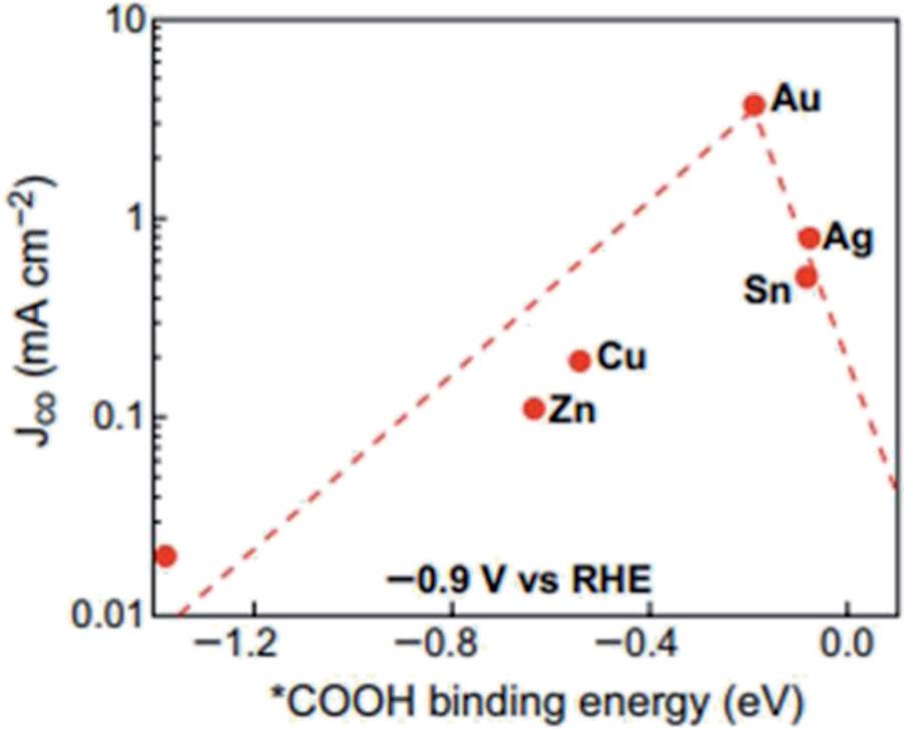
Volcano plot of different metals towards the electrochemical reduction (ECR) of CO_2_. Reprinted with permission from ref. 43. Copyright 2017, American Chemical Society.

One of the most effective ways to reduce the cost of Au catalysts is by using nanoparticles (NPs). In the study conducted by Mistry *et al.*, they found that the catalytic activity in terms of current density was improved significantly when the size of Au NPs was reduced from 7.7 nm to 1.1 nm over a wide potential range, as seen in Fig. 4.^28^ However, this resulted in a lower FE for the formation of CO with the FE of H_2_ increasing. This was ascribed to the reduction in the size of the Au NPs, which led to an increase in the abundance of both edge and corner sites. The edges were theorized to enable the formation of CO, while the corners were the key sites for the formation of H_2_.^28^ Therefore, although the overall ECR performance of Au NPs for CO increased as the particle size became smaller, the lower FE of CO was not ideal. This theory was also supported by Zhu *et al.*, who observed the same trend in their study on Au NPs and confirmed their observation by improving the FE of CO from 90% to 97% by embedding the Au NPs in an ionic matrix to prevent the HER reaction.^44^ Furthermore, their DFT calculations supported both Mistry *et al.* and their hypothesis that the edges promote CO evolution, while the corner sites are active for H_2_ evolution. Interestingly, in a follow up work by Back *et al.*, their DFT study on Au NPs showed contradictory results, where their findings suggested that the corners promote CO evolution, while edges are for H_2_ evolution.^45^ They attributed the discrepancy in their findings in comparison to Zhu *et al.* to the quantum size effect, given that their modelling was achieved using large Au NPs (309 atoms or more), which would give more accurate results. The quantum size effect can be defined as strong binding energies as a result of the size of the Au NPs. In the case of Au NPs with size less than 2 nm, the sizing model was affected. Therefore, for correct prediction of Au NPs, this theorized quantum size effect should be considered to acquire Au NPs with the optimal size, which is strongly correlated with their catalytic activity.

**Fig. 5:**
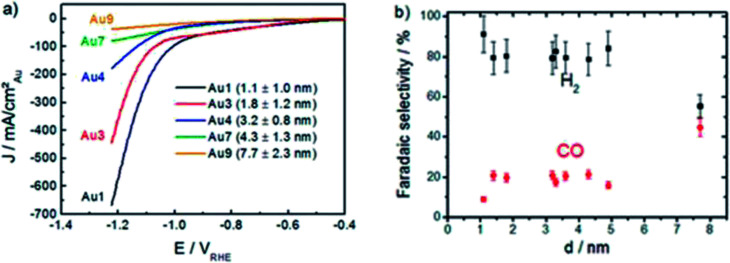
(a) Linear sweep voltammetry (LSV) curves of the ECR of CO_2_ on gold (Au) nanoparticles (NPs) of various sizes. (b) Effect of Au NP size on the faradaic efficiency (FE). Reprinted with permission from ref. 28. Copyright 2013, American Chemical Society.

Another way to improve the catalytic performance of Au NPs is by shape modification. In a recent study conducted by Yang *et al.*, they found with DFT calculations that the (2 2 1) facet of Au had a lower potential barrier compared to the traditional (1 1 1), which was the focus of studies in the past, as seen in Fig. 5.^46^ The size effect of the Au trisoctahedron (TOH) NP was found to have a direct correlation with the faradaic efficiency (FE), where an increase in particle size to more than 100 nm caused the FE for CO to decrease by more than 60%. The SEM image of the 50 nm Au TOH also showed the clear edge features of the NP, which improved its performance for the electrocatalytic reduction of CO_2_. Considering this, they designed and synthesized Au trisoctahedron for the ECR of CO_2_ to CO. They discovered that Au TOH could increase the FE for CO by 1.5 times compared to colloidal Au. Furthermore, they found that the performance of the Au TOH decreased with an increase in particle size. They hypothesized that with an increase in the Au TOH particle size, there was a systematic decrease in the edge to face ratio, which resulted in a decline in the performance of Au TOH given that the edge sites of Au TOH were found to be the most active catalytic sites due to their good balance between COOH stability and CO desorption free energy.

In conclusion, Au NPs are theoretically the best electrocatalyst for the ECR of CO_2_ to CO. Recent studies have explored different ways such as shape and morphology manipulation to control and improve their catalytic activity. However, their underlying mechanism is still under debate, with both Zhu and Back *et al.* both showing contradictory DFT results. Furthermore, the optimization to improve the cost efficiency by reducing the size of Au NPs has not yielded favorable results. Therefore, there is still much need for further research in both mechanism study and optimization for Au to be deemed a suitable candidate for the large-scale ECR process.

#### 3.1.2 Silver (Ag)

Ag is another highly investigated potential electrocatalyst for ECR. According to Fig. 3, it is the second ideal electrocatalyst for the ECR to CO process. This is due to similar reasons to that of Au, where it has the second optimal binding for the COOH intermediate, leading to an overall lower overpotential. In fact, theoretically, its required overpotential is lower than that of Au but at the cost of current density. Again, similar to that of Au, Ag is also expensive although significantly cheaper than Au. Therefore, researchers have been looking for ways to reduce its cost as well.

**Fig. 6:**
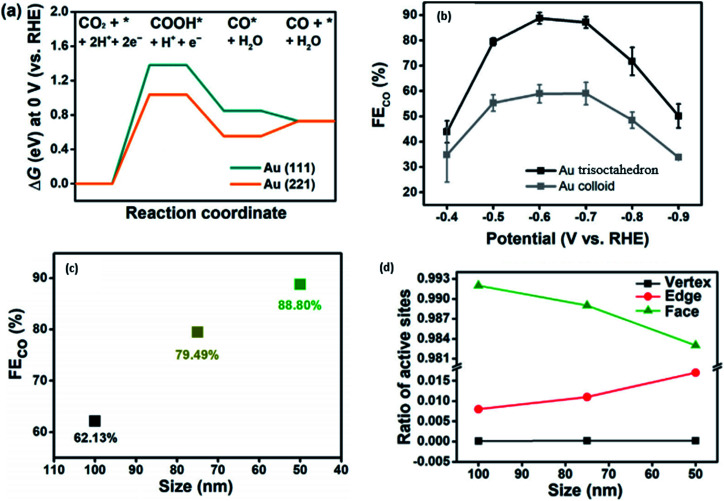
(a) Gibbs free energy diagram for the ECR of CO_2_ to CO on Au (1 1 1) and Au (2 1 1). (b) FE of Au trisoctahedron (TOH) compared to that of Au NPs (colloid). (c) Effect of the size of Au TOH on FE. (d) Ratio of active sites in Au TOH with respect to particle size. Reprinted with permission from ref. 46. Copyright 2020, Elsevier.

Similar to Au, researchers have tried to improve the performance (to enhance its cost benefit) of Ag by using NPs with modification of their size. Both Salehi-Khojin *et al.* and Kim *et al.* found that Ag NPs performed better in terms of current density and FE for the ECR of CO_2_ to CO compared to bulk Ag films.^47,48^ They both reported that they found 5 nm to be the optimal size for the best activity by Ag NPs, with Salehi-Khojin *et al.* reporting a 10-times increment in activity, while Kim *et al.* reported a 4.8-times enhancement with reference to Ag film/foil, as seen in Fig. 6. Interestingly, both groups reported different reasons for this enhancement. Salehi-Khojin *et al.*, using DFT modelling, found that reducing the Ag NPs size below 5 nm resulted in an increase in the binding energy of the COOH intermediate and decrease in the rate of the desorption of OH, which reduced the overall reaction rate. This is the typical behaviour shown by the volcano plot. Kim *et al.* attributed their enhanced performance to the cysteamine anchoring agent. According to their DFT calculations, they found that the cysteamine anchoring agent led to the localization of unpaired electrons on the surface of the Ag NPs, which helped to stabilize the key COOH intermediate. Regarding the optimal performance of their 5 nm Ag NPs, they attributed this to the optimal coverage of the anchoring agent on the Ag NPs, with other sizes resulting in suboptimal coverage (too much or too little coverage).

**Fig. 7:**
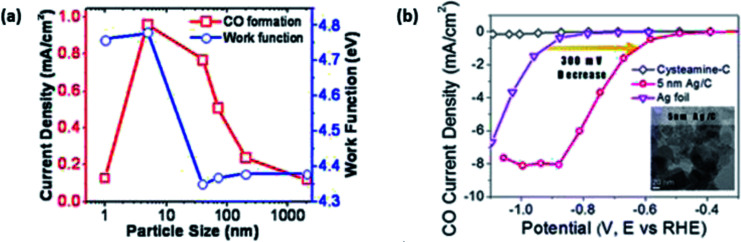
(a) Graph depicting the current density and work function against the particle size of silver (Ag) NPs. Reprinted with permission from ref. 47. Copyright 2012, American Chemical Society. (b) Linear sweep voltammetry (LSV) curves of 5 nm Ag NPs compared to those of Ag foil and cysteamine-C. Reprinted with permission from ref. 48. Copyright 2015, American Chemical Society.

However, in the recent study conducted by Back *et al.*, their DFT calculations discovered that in contrast to Au, the Ag NP edges are the most active sites for the ECR of CO_2_ to CO.^45^ They also found that unlike Au, the optimal size of Ag NPs was 2 nm with minimal interference from the unwanted and competing HER process. This was due to the increase in highly active sites, as seen in Fig. 7. It is also worth noting that Back *et al.* collaborated their findings with the experimental findings observed by Salehi-Khojin *et al.* They explained that in their calculations, they did not consider the quantum-size effect. Therefore, the trend observed in the Ag NPs by Salehi-Khojin *et al.* from 1–5 nm was not consistent with their results. However, for the 5–200 nm Ag NPs, the quantum-size effect was not observed, which is consistent with the findings by Salehi-Khojin, where the edge sites were the most active.

**Fig. 8:**
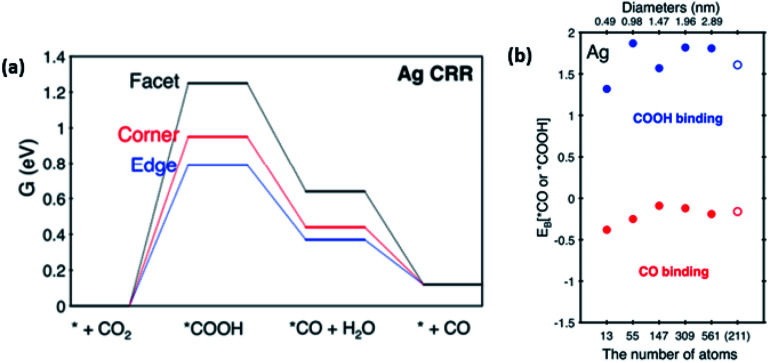
(a) Gibbs free energy diagram on different facets of silver (Ag) NPs for the electrochemical reduction (ECR) of CO_2_. (b) Binding energies of Ag NPs of different sizes at the (2 1 1) edge. Reprinted with permission from ref. 45. Copyright 2015, American Chemical Society.

**Fig. 9:**
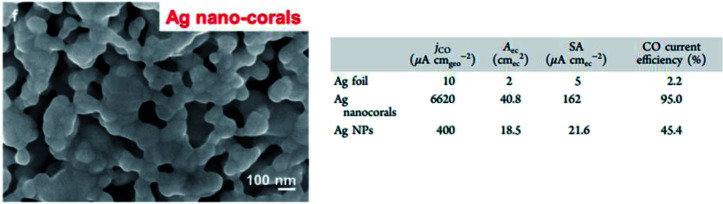
Ag nano-corals with high catalytic activity (38 times better than Ag foil). Reprinted with permission from ref. 49. Copyright 2015, American Chemical Society.

There have also been attempts to increase the performance of Ag NPs by using a variety of different shapes. Hsieh *et al.* used high surface area Ag nanocoral to improve the performance of Ag NPs.^49^ As seen in Fig. 8, they reported a 32-fold increment in catalytic activity compared to bulk Ag foil. This enhancement was ascribed to both the nanostructure morphology effect, and importantly the presence of doped chlorine, which inhibited the undesirable HER side reaction. In the recent study by Liu *et al.*, they used Ag nanocubes to achieve this effect.^50^ They reported a near unity FE (99%) for Ag nanocubes with a length below 25 nm (L25-Ag-NCs), exhibiting an ultra-low overpotential (146 mV *vs.* RHE), higher energy efficiency (64%) and durability (stability over 18 h) compared to nanocubes with a length of less than 70 nm, Ag NPs and Ag foil, as seen in Fig. 9. They substantiated their results with DFT calculations, which showed that in L25-Ag-NCs, the maximum number of energetic favored sites were introduced through precise synthesis of the nanostructure enclosed by the Ag (1 0 0) facet.

In summary, Ag is seen as a promising alternative to Au, being theoretically the second best electrocatalyst for the ECR of CO_2_ to CO. Interestingly, unlike Au, Salehi-Khojin and Kim *et al.* showed that reducing the size of Ag NPs could significantly improve both their catalytic performance and product selectivity although the former attributed the increase to the stabilization of the COOH intermediate, while the latter hypothesized that the cysteine anchoring agent was key in the synthetic process. Overall, this shows the potential of lowering the cost of Au-based electrocatalysts by improving the mass activity. Other groups such as Hsieh and Liu *et al.* were successful in improving the performance of Ag by its shape manipulation (coral and cubes respectively). Lastly, the DFT calculations by both Salehi-Khojin and Back *et al.* are in agreement with the edges being the key factor for CO evolution, which can be targeted in the future for further optimization. Hence, Ag has been shown to be a cheaper alternative compared to Au with the possibility of significantly higher mass activity due to the trend of improved electrocatalytic activity with a decrease in its size.

#### 3.1.3 Palladium (Pd)

Next, Pd is an emerging element at the centre of recent advancements for the ECR of CO_2_ to CO due to its ability to selectively pick either CO or HCOO^−^ as its major products. This can be established by simple modifications to either the applied potential or by introducing heteroatomic doping.

**Fig. 10:**
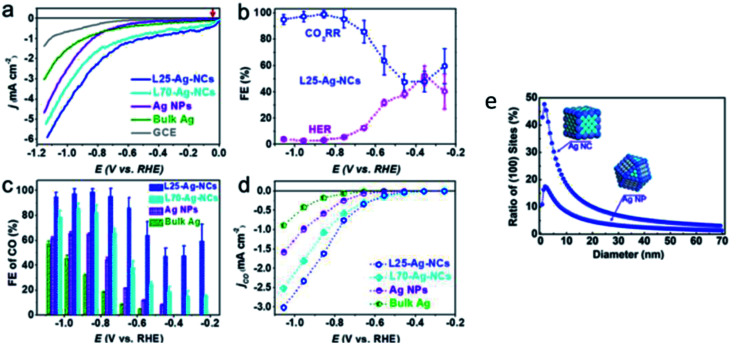
(a) Linear sweep voltammetry (LSV) curves of varying sizes of silver (Ag) nanocubes (NC) compared to those of Ag NPs, bulk Ag and GCE. (b) Overall FE of 25 nm Ag-NCs. (c) FE of CO and (d) normalized LSV curves for 25 and 75 nm Ag-NCs, Au NPs and bulk Ag. (e) Ratio of (1 0 0) active sites of Ag-NC compared to that of Ag NPs. Reprinted with permission from ref. 50. Copyright 2020, American Chemical Society.

In previous studies on Pd, it was found by Gao *et al.* that the final product of either CO or HCOO^−^ could be actively determined by the type of Pd surface present, as seen in Fig. 10.^51^ Based on their *in situ* XPS and DFT results, they found that with the hydrogen-absorbed Pd surface over a mixture of α- and β-phase palladium-hydride (PdH) core, the formation of HCOO^−^ was more likely *via* the HCOO* intermediate step at an FE as high as 98%. Alternatively, when the β-phase PdH core was covered with a metallic Pd surface, CO was the major product of the ECR with an FE of 93.4%. This was due to the fact that the formation of CO was promoted *via* the COOH* intermediate instead.

**Fig. 11:**
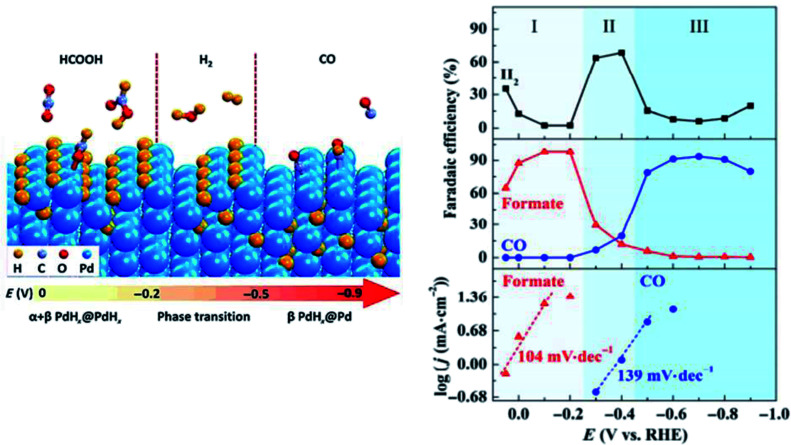
The effect of different phases of palladium (Pd) on the product of the electrochemical reduction (ECR) of CO_2_. On the β-phase of the palladium–hydrogen (PdH) core, up to 93.4% faradaic efficiency (FE) towards carbon monoxide (CO) can be achieved. Reprinted with permission from ref. 51. Copyright 2017, Springer.

However, in the recent study conducted by He *et al.*, they found that the PdH core, which was previously determined to be essential for the formation of the Pd NP catalyst, was not optimal for the ECR process.^52^ In their study, as seen in Fig. 11, they discovered that the Pd–N_4_ site on a nitrogen-doped carbon-supported Pd single atom (Pd-NC) was more suitable for the ECR of CO_2_ to CO. It was shown *via in situ* XAFS and DFT calculations that the Pd–N_4_ single atom site can stabilize the formation of the CO_2_ intermediate, therefore enhancing the electrocatalytic performance of the Pd catalyst at low overpotentials. This translated into a higher mass activity performance of 373.0 mg_Pd_^−1^ for Pd-NC compared to 28.5 mg_Pd_^−1^ for the traditional Pd/C. Compared to the active phase engineering conducted by Gao *et al.*, the performance of the Pd-NC was better at a lower potential but suffered at higher overpotentials.

**Fig. 12:**
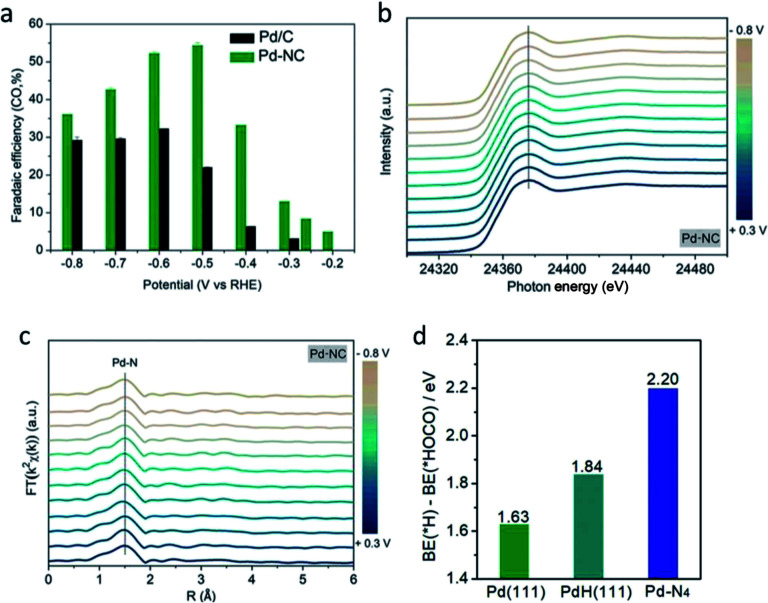
(a) faradaic efficiency (FE) towards CO for Pd-NC and Pd/C, (b) XANES spectra and (c) Fourier-transformed *k*^2^-weighted EXAFS profiles of Pd-NC. (d) DFT calculation of the difference in the binding energy of H and HOCO on Pd (1 1 1), PdH (1 1 1) and Pd–N_4_. Reprinted with permission from ref. 52. Copyright 2020, Wiley.

Lastly, in the recent work by Dong *et al.*, they used facet design to enhance the ECR performance of Pd NPs.^53^ As seen in Fig. 12, Dong *et al.* synthesized Pd NPs with an equal size but different shapes to represent different facets, including concave cubes, cubes and octahedrons to represent the (3 1 0), (1 0 0) and (1 1 1), respectively. Based on both experimental results and DFT calculations, they found that the concave cubes enclosed with (3 1 0) facet had the best ECR activity with an FE selectivity of 90.6% compared to the octahedrons and cubes with an FE of 71.3% and 68.2%, respectively. This result is in good agreement with the results reported by Gao *et al.* They further found that the reason for the high activity of the (3 1 0) facet was due to the stabilization of the formed COOH* intermediate and the ease of desorption of CO* from the (3 1 0) facet.

In summary, recent advances in Pd electrocatalysts have been established *via* core and surface manipulation. Researchers such as Dong *et al.* found success in facet design. However, in terms of the design and understanding of optimal Pd NPs, there has been some contention with Gao *et al.* finding the Pd–H core with pure Pd metal covering its surface to have the optimal results, while He *et al.* disagreed with their findings, reporting a single Pd atom core with Pd–N_4_ active surface site having overall better catalytic and mass activity. Also, the current price of Pd metal is 70 600 USD, which is 28% higher than Au metal and significantly greater than the cheaper Ag metal of 610 USD. Therefore, although Pd is a potential electrocatalyst for the ECR process, there is still much to be understood in terms of mechanism and electrocatalyst design before it can be utilized effectively.

### 3.2 Non-noble metals

For non-noble metals, zinc (Zn) and bismuth (Bi) are two of the more popular metals, which are actively studied currently. The reason for this is different for each metal and will be discussed in greater detail below.

#### 3.2.1 Zinc (Zn)

Zn has been widely regarded as a potential alternative catalyst to Au, Ag and Pd due to its low cost and non-toxic properties. However, more research has to be conducted on this metal for it to be a viable replacement for the above-mentioned electrocatalysts.

**Fig. 13:**
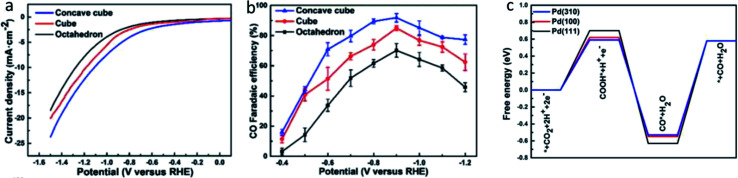
(a) Linear sweep voltammetry (LSV) curves and (b) CO FE of concave cube, cube and octahedron Pd NPs, which are Pd (3 1 0), (1 0 0) and (1 1 1), respectively. (c) DFT calculations of the free energy diagram for the ECR of different facets of Pd NPs. Reprinted with permission from ref. 53. Copyright 2019, Elsevier.

Quan *et al.* fabricated Zn nanoplates (n-Zn) by electroreducing Zn foil, as seen in Fig. 13.^54^ n-Zn exhibited superior ECR properties compared to Zn foil. Furthermore, they also found that the Zn electrocatalyst generally performed better for the ECR of CO_2_ to CO in NaCl solution compared to NaHCO_3_ solution. Their n-Zn had an FE of 93% in NaCl compared to that of 60% in NaHCO_3_ over a 10 h operation. They proposed that the reason for this improvement was due to the fact that the Cl^−^ ions in the NaCl solution could greatly suppress the competing HER process. Consequently, this promoted the formation of the CO_2_˙^−^ radical, which enhanced the conversion efficiency of CO_2_, as illustrated in Fig. 13. This trend was also observed when they used Ag NPs instead, with the FE of the Ag NPs increasing from 20% to 70% by simply changing the solution from NaHCO_3_ to NaCl.

**Fig. 14:**
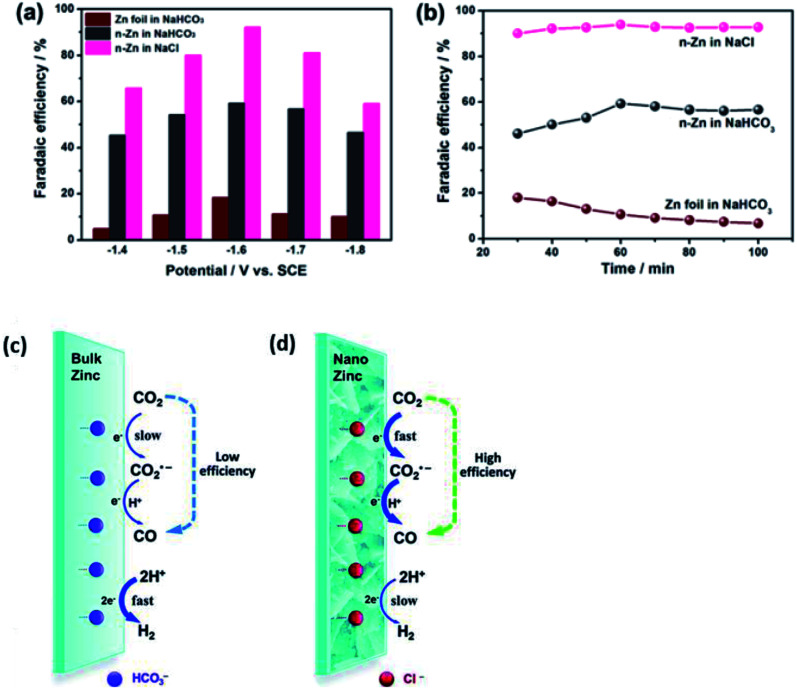
Comparison between zinc (Zn) foil and n-Zn electrodes in CO_2_ reduction. (a) FE_CO_*versus* applied potential. (b) FE_CO_*versus* electrolysis time at −1.6 V. Schematic illustration of CO_2_ reduction to CO on Zn electrodes in NaHCO_3_ (c) and NaCl solution (d). Reprinted with permission from ref. 54. Copyright 2015, Royal Society of Chemistry.

In more recent works, the focus has been increasing the performance of Zn electrocatalysts by using porous Zn, which possessed an increased surface area. In the study by Lu *et al.*, they electrodeposited Zn(NO_3_)_2_ on a Zn film, which resulted in a highly porous Zn network, as seen in Fig. 14.^55^ They found that the roughness of the electrodeposited porous Zn network (ED-Zn) was correlated with the specific capacitance and FE. The roughest ED-Zn with a roughness factor of 17.4 had the highest FE of 80% at −1.1 V *vs.* RHE.

**Fig. 15:**
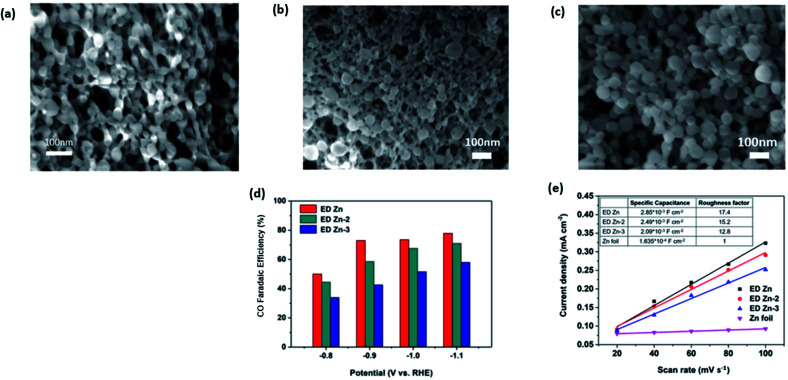
SEM images of (a) ED Zn, (b) ED Zn-2 and (c) ED Zn-3 and (d) FE of CO on ED Zn, ED Zn-2 and ED Zn-3 at various constant potentials ranging from −1.1 to −0.8 V *vs.* RHE. (e) Specific capacitances and roughness factors of ED Zn, ED Zn-2, ED Zn-3 and Zn foil. Reprinted with permission from ref. 55. Copyright 2018, Elsevier.

A more in-depth study was conducted by Luo *et al.*, where they observed an increased ECR performance for porous Zn.^56^ In their study, they electrodeposited Zn on Cu mesh to obtain a porous Zn network, as shown in Fig. 15. Their porous Zn (p-Zn) exhibited an excellent FE of 95% at −0.95 *vs.* RHE. They attributed this superior performance to the local pH effect, where p-Zn had a higher geometric current density compared to Zn foil, which led to a higher proton consumption rate near the electrode, resulting in the suppression of the competing HER. This was supported by their experiment, where they tested p-Zn in buffers with various buffer strengths and found that the FE of p-Zn improved dramatically when the local pH increased. Therefore, they emphasized the importance of both surface area and the impact of local pH to improve the ECR performance of Zn.

In conclusion, Zn is a promising electrocatalyst for the ECR process due to its low cost. Recent advancements have targeted the shape of Zn (nanoplatelets and porous) to improve its performance. However, there is a lack of understanding on the underlying mechanism in the Zn ECR process. There has been some attempt to understand this, with Xiao *et al.* reporting that the (1 0 0) facet and edge sites are optimal for the ECR to CO but their findings were limited to hexagonal Zn nanoplatelets.^57^ Therefore, additional studies on more generic Zn NPs are needed before Zn can be applied in the ECR process.

#### 3.2.2 Fe- and Co-based single-atom catalysts (SAC)

Single-atom catalysts (SAC) have been established as emerging electrocatalysts for CO_2_ conversion to CO primarily owing to their excellent catalytic activity.^58^ In the study by Peng *et al.*, metal-based SAC such as Fe-based were shown to be viable alternatives to the noble metal-based electrocatalysts. Cobalt (Co)-based SAC also have a high current density even though it is comparatively lower than that of Fe-based SAC. However, there are limitations associated with the use of metal-based SAC. For instance, the method to obtain SAC requires the precisely controlled removal of the metallic nanoparticles (NPs). Also, the agglomeration of the NPs with molecular moieties disrupts the catalytic reactivity of the system. Given that demetallation occurs primarily for Fe-based SAC, other non-noble metals such as Co and zinc have been studied as an alternative source to produce non-noble metal-based single-atom electrocatalysts as complement or viable alternative to electrocatalysts.

Hu *et al.* also performed an extensive study on the use of Fe, Co and Ni, which are categorized as transition metals, and found to have great significance to the catalytic activity.^74^ Not only the catalytic activity was tremendously enhanced, but also the high selectivity for the products was retained. Interestingly, Ni-doped carbon has been established to have an FE as high as 93%. All these metals have a huge correlation with the formation of products that boost the CO_2_RR activity, while retaining the porosity of carbon electrocatalysts.

#### 3.2.3 Bismuth (Bi)

Bi is a potential electrocatalyst that is both cheap and environmentally friendly. However, Bi is more commonly used for the ECR of CO_2_ to HCOOH instead of CO, especially in aqueous electrolyte. Nevertheless, when the electrolyte is changed to an ionic liquid, the main product of the ECR process using Bi changes from HCOOH to CO. Therefore, Bi has potential as an alternative electrocatalyst to Au, Ag and Pd.

**Fig. 16:**
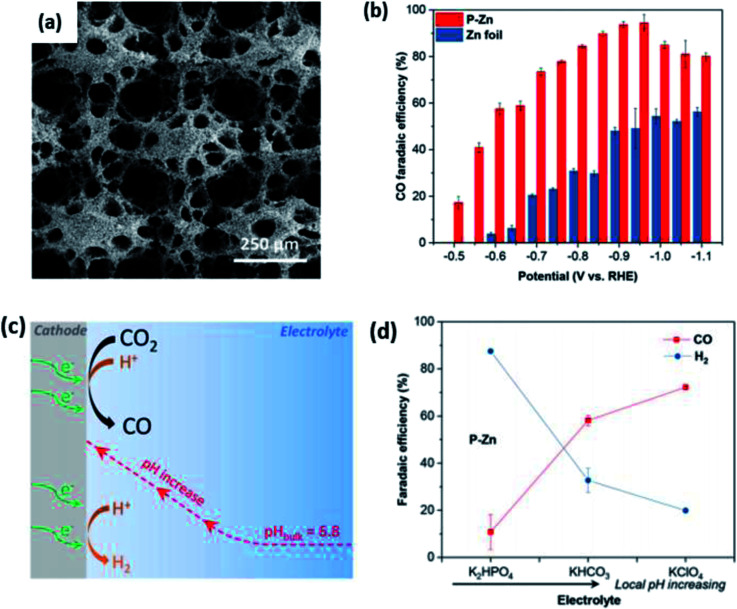
(a) SEM image of porous electrodeposited Zn on Cu mesh (P–Zn). (b) CO FE of P–Zn and Zn foil. (c) Schematic illustration of the local pH effect on the ECR of CO_2_. (d) FE of P–Zn in different electrolytes. Reprinted with permission from ref. 56. Copyright 2019, American Chemical Society.

In the earlier study conducted by DeMeglio *et al.*, they electrodeposited Bi on a glassy carbon substrate, as seen in Fig. 16.^59^ They found that the Bi electrocatalyst they synthesized exhibited an FE as high as 95% in 1,3-dialkyl-substituted imidazolium-based ionic liquids (commonly used in carbon sequestration) with almost no activity without the use of the ionic liquids. They theorized that the enhancement was the result of the possible interaction between the Bi^0^ and Bi^3+^ sites that stabilizes the key CO_2_˙^−^ intermediate. However, the role of the ionic liquid is still unclear, where it is suggested that the ionic liquid may act as a mediator or there may be an interaction between the CO_2_ at the bulk Pt electrodes and imidazolium cations, which can enhance the CO_2_ electroreduction.

**Fig. 17:**
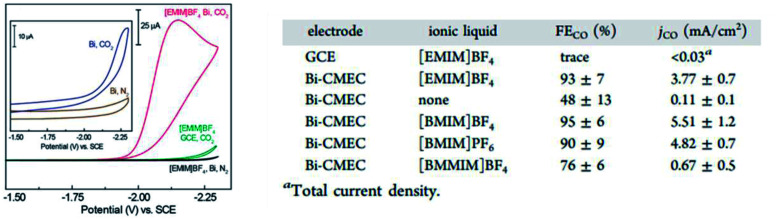
CV traces recorded for Bi-modified and bare GCEs in MeCN containing 20 mM [EMIM]BF_4_. Inset: Bi-modified GCE in MeCN without ionic liquid (IL). Table summarizing the faradaic efficiencies (FE_CO_) and current densities (*j*_CO_) for the ECR of CO_2_ to CO at an applied potential of −1.95 V *vs.* SCE in MeCN. Reprinted with permission from ref. 58. Copyright 2013, American Chemical Society.

In another study conducted by Zhang *et al.*, they reported similar results using Bi NPs in acetonitrile, which was mediated using 1-butyl-3-methylimidazolium trifluoromethanesulfonate ([bmim][OTf]).^60^ They found that the size of the Bi NPs played a significant role in the ECR of CO_2_ to CO with a decline in the FE from 87% to 69% as the particle size decreased, as seen in Fig. 17. However, after surface activation *via* hydrazine treatment (reduction), the particle size had no effect, with both sizes of Bi NPs exhibiting almost similar FE. They speculated that the reason why the size effect was no longer present is due to the direct interaction (proton transfer) between the ionic liquid and the adsorbed CO_2_, which means that the proton transfer step is no longer affected by the morphology of Bi.

Briefly, Bi has been considered a viable electrocatalyst for the ECR to CO because it is cheap and environmentally friendly. Research has shown both the high current density and FE with the synthesis process (surface activation) and the type of ionic liquid used are the key factors. However, there is still a lack of understanding on the roles played by ionic liquids in the ECR of CO_2_ to CO in Bi. This is related to another obvious problem, where Bi is still currently limited to organic solvents and is unstable for use in aqueous solvents. The sizing effect of the Bi NP is more prevalent in organic solvents due to the lack of protons for the reduction of the metal-based oxides. Alternatively, in aqueous solvents, where a higher proton number is present, the sizing effect of the Bi NP is less dominant. Also, this render the aqueous solvents less stable in terms of sizing effect compared to organic solvents, where the sizing effect is more dominant.^60^ Thus, by discovering the key role that the ionic liquid plays, Bi can be eventually considered as a potential candidate for the ECR process.

### 3.3 Non-metal-based electrocatalyst

#### 3.3.1 Carbon nanomaterials

For non-metal-based electrocatalysts, carbon nanomaterials are a recent popular alternative. Carbon nanomaterials traditionally serve as a support material for the efficient loading of the active metal electrocatalyst. Recently, researchers have started to focus on various methods of activating these carbon nanomaterials for their use as electrocatalysts instead due to their low cost. These carbon nanomaterials can be separated into 3 categories, including 0D, 1D and 2D nanomaterials.

**Fig. 18:**
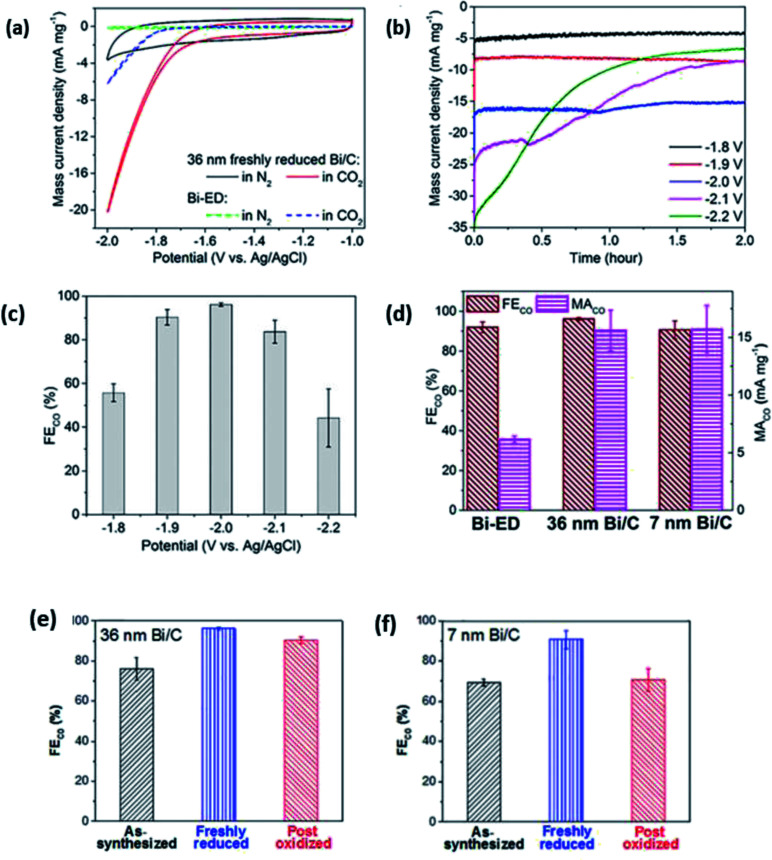
(a) Cyclic voltammograms of 36 nm freshly reduced Bi/C and Bi-ED in MeCN containing 100 mM [bmim][OTf]. (b) Current density transients and (c) FE_CO_ under constant potential electrolysis during CO_2_ reduction at different applied potentials. (d) FE_CO_ and MACO on Bi-ED (36 nm and 7 nm freshly reduced Bi/C). FE_CO_ of 36 and 7 nm Bi/Cs under different treatment. Reprinted with permission from ref. 59. Copyright 2016, American Chemical Society.

**Fig. 19:**
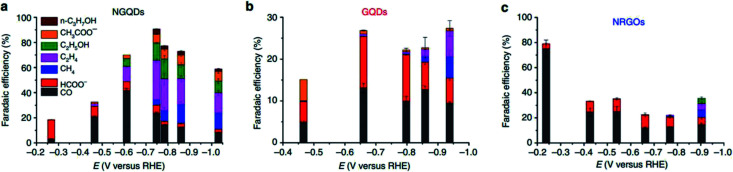
FE of different products of (a) NGQDs, (b) GQDs and (c) NRGOs. Reprinted with permission from ref. 60. Copyright 2016, Nature Research.

In the case of 0D carbon nanomaterials, graphene quantum dots (GQD) are usually used. GQD are atom-thin (less than 2 nm in thickness) and have minuscule transverse directions (less than 10 nm). In the research conducted by Wu *et al.*, they found that by doping GQD with nitrogen (NGQD), they could improve the FE of the ECR of CO_2_ to CO from 13% to 43%, as seen in Fig. 18.^61^ They speculated that this improvement in selectivity was due to the heteroatomic N doping and high exposure of the edge sites of the NGQD but did not present any investigation into this. In a subsequent study by Zou *et al.*, their modelling found that the pyridinic N of NGQD enhanced their bonding with the key COOH* intermediate, which in turn actively promoted the ECR of CO_2_ to CO, as seen in Fig. 19.^62^ Therefore, they concluded that heteroatomic N doping can play a key role in the future design and application of 0D carbon nanomaterials.

**Fig. 20:**
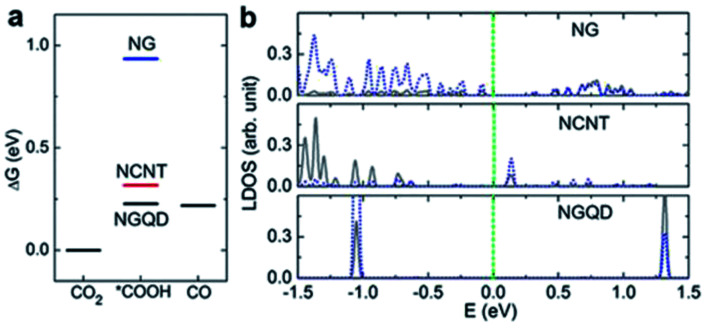
(a) Thermodynamic free energy diagram for the conversion of CO_2_ to CO on different N-doped carbon materials, with adsorbed *COOH as the intermediate state. (b) LDOS for pyridinic N (blue dotted lines) and C atoms (gray lines) from adsorbed *COOH. These two atoms form N–C bonds in the intermediate states. Reprinted with permission from ref. 61. Copyright 2017, Nature Communications.

In the case of 1D carbon nanomaterials, carbon nanotubes (CNT) are the carbon allotropes that represent this group. In an interesting study by Ma *et al.*, they applied the same principle by heteroatomically doping CNT with nitrogen (NCNT).^63^ They reported that their NCNT could maintain an FE of CO above 94.5% between −0.6 to −0.9 V *vs.* RHE over a 40 h long electrolysis process, as seen in Fig. 20. They also found that the pyridinic N was the key factor in their high performance with speculation that there may be weak interaction between CO_2_ and pyridinic N, which aids in the formation of CO. Lastly, they also reported the use of NCNT for gas-phase CO_2_ electrolysis, which could actively suppress the competing HER reaction with the FE of CO kept at nearly 100% over a 17 h electrolysis. This shows that gas-phase electrolysis is a potential approach to maximize the efficiency of CO production with easier industrial translation.

**Fig. 21:**
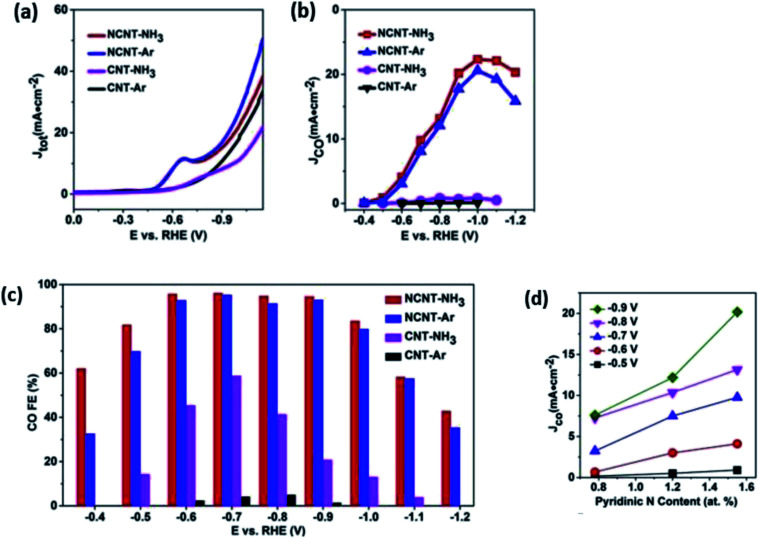
(a) Linear sweep voltammetry (LSV) curves for catalysts in CO_2_-saturated 0.5 M NaHCO_3_ aqueous solution at 50 mV s^−1^. (b) Partial current density of CO *vs.* applied potential for the catalysts. (c) CO FEs at various applied potentials. (d) CO current density at different applied potentials *vs.* the content of pyridinic N (at%) Reprinted with permission from ref. 62. Copyright 2019 Elsevier.

**Fig. 22:**
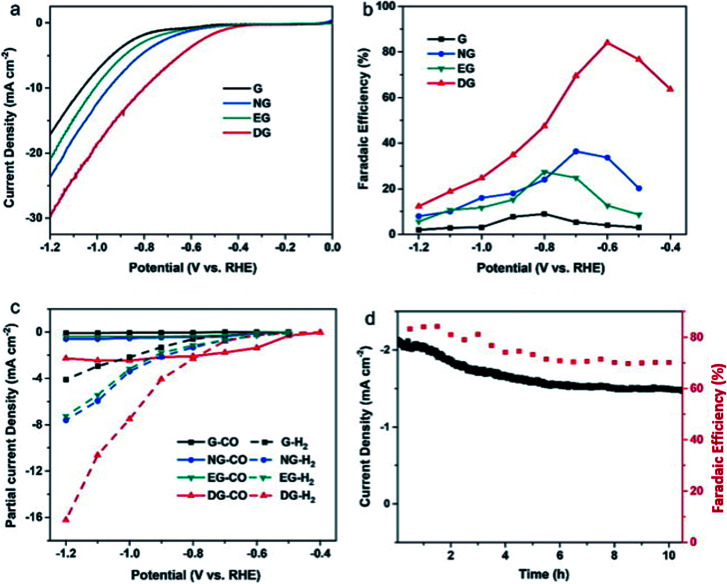
(a) Linear sweep voltammetry (LSV) curves for pristine graphene, nitrogen-doped graphene (NG), edge-filled graphene (EG) and distorted graphene (DG) in CO_2_-saturated 0.1 M KHCO_3_ electrolyte. The scan rate was 10 mV s^−1^. (b) Faradaic efficiencies to CO at different applied potentials on graphene, NG, EG and DG, respectively. (c) Partial current densities of CO and H_2_ generated on graphene, NG, EG and DG. (d) Time-dependent total current density curve (left *y*-axis) and faradaic efficiency (right *y*-axis) for CO of DG in CO_2_-saturated 0.1 M KHCO_3_ solution at an applied potential of 0.6 V *vs.* RHE for >10 h. Reprinted with permission from ref. 63. Copyright 2019, Elsevier.

Finally, for 2D carbon nanomaterials, graphene is the representative for this group. Han *et al.* recently performed a study comparing the effects of nitrogen-doped graphene (NG) with other sources of graphene such as regular graphene (G), edge-filled graphene (EG) and defective graphene (DG), as seen in Fig. 21.^64^ They reported surprising results, where DG had the best performance among the different types of graphene. DG had the highest FE of CO at 84% over a 10 h period. They theorized that this superior result was due to the synthesis process, where the DG was synthesized by the removal of nitrogen in NG. The defects caused by nitrogen doping and subsequent removal of the doped nitrogen were special and resulted in unique defect sites, which were not replicated in EG (plasma process) (Fig. 22).

In summary, carbon nanomaterials are an inexpensive alternative compared to metal-based electrocatalysts for the ECR of CO_2_ to CO. Unfortunately, carbon nanomaterials are generally inert and need to be activated before they can be utilized as an electrocatalyst. The literature shows the current trend for this activation across all varieties of carbon nanomaterials by the nitrogen doping. However, this gives rise to another issue where the activation process is achieved *via* hydrothermal treatment (typically), which increases the production cost. Besides, the performance of carbon electrocatalysts is subpar in terms of both catalytic performance and FE compared to their metal counterparts. The one redeeming factor that carbon electrocatalysts have is their enhanced durability (albeit overall lower), which attributed to their inert nature. Therefore, for carbon nanomaterials to be used effectively over metal-based electrocatalysts, extensive studies are still required to improve both their catalytic performance and FE.

#### 3.3.2 Electrocatalyst advances

The CO_2_RR has achieved much progress owing to the studies on transition metal chalcogenides (TMCs) as catalysts, which enhanced the selectivity of products through the established effective TMC catalysts, as shown in the study by Gao *et al.*^65^ The conversion of CO_2_ to CO with heterogeneous catalysts in the investigation by Zheng *et al.* showed selective CO conversion with improved catalytic capabilities toward single-atom catalysts, which were discussed in an earlier section.^66^ The design of electrocatalysts was discussed in greater detail in the study by Gao *et al.*, where electrocatalysts were studied for their reduction capability, and therein the enhanced current density of the catalysts. These design principles have been found to have a great influence on the catalytic activity of the electrocatalysts.^67^ Alternatively, the study by Kibria *et al.* showed mechanism models for the design of electrocatalysts with the catalytic capabilities that are suitable for economic productivity.^68^

## 4. Conclusion and future prospects

In this review, we summarized the latest developments in nano-based electrocatalysts for the ECR of CO_2_ to CO. Noble metals, non-noble metals and carbon-based electrocatalysts were discussed and their advantages and problems highlighted.

In the case of noble metals, Au, Ag and Pd were the focus in this review. Au is theoretically the best electrocatalyst for the ECR of CO_2_ to CO because it has optimal binding energy for the key intermediate of COOH. This has translated to it having the highest catalytic activity and FE among the catalysts. However, Au suffers from poor mass activity due to the competitive HER during the optimization, which hampers its performance. Therefore, new ways to optimize the mass activity of Au are needed. Alternatively, Ag is theoretically the second best electrocatalyst. Its overall performance is inverse to that of Au. Researchers have shown the higher mass activity of Ag due to the lower HER as its size is reduced. Unfortunately, Ag has a lower current density, and in some cases lower FE compared to Au. Next, Pd has been the focus of recent research interest due to its high selectivity for the products. Regrettably, the exact mechanism behind the high performance of Pd is still under debate, with studies claiming the PdH core with pure Pd on its surface is key, while others have contested these claims with their own results, showing that the use of a pure Pd core with Pd–N_4_ active sites is better. Therefore, further studies on Pd are still needed before it can be actively used as an electrocatalyst. Overall, noble metal electrocatalysts have the greatest potential of being adapted and used for industrial applications.

For non-noble metals, Zn and Bi have been evaluated as the top choices in this field. In particular, Zn is cheap and has shown great potential as a suitable replacement for noble metals. Although new developments have shown that the performance of Zn is almost comparable with that of Ag, it still has a debilitating problem where its performance is heavily influenced by the local pH effect. This severely affects the potential application of Zn given that the local pH effect is difficult to control on a macro-scale. Therefore, new or improved optimization is needed for Zn to be considered as a suitable electrocatalyst. Regarding Bi, it has been shown as cheap and environmentally friendly alternative. Bi has also shown the highest FE among the various catalysts. However, these results are only limited to ionic liquids and when it is exposed to aqueous solvents, unwanted side reactions occur. Thus, studies are required to establish the understanding on the usability of enhanced Bi in aqueous solvents before it can be used effectively.

Lastly, for carbon-based electrocatalysts, they are the cheapest electrocatalysts compared to the rest. New research has shown both in terms of theoretical modelling and experiments that nitrogen doping is the most effective method for improving the otherwise inert but cheap carbon electrocatalysts. This was found to be true for 0D, 1D and 2D carbon catalysts. However, their performance in terms of current density and FE is still inadequate compared to traditional metal catalysts. Nevertheless, one key advantage that carbon-based electrocatalyst have is their durability and resistance to poisoning over time, which are superior to that of their metal counterparts.

Here, we highlight future prospects that researchers can explore.

### 4.1 Amorphous materials

Recent developments in other catalysis fields have shown the potential of amorphous catalysts. Amorphous structures provide abundant highly active sites, which may be useful for the ECR process and should be explored in depth.

### 4.2 Bi-metallic/tri-metallic

Researchers have shown promising results by combining different metals with greater advantages, overcoming the shortcomings of the individual catalysts. Some of these studies have been done on the ECR process but are limited to high-order products such as CH_3_OH. Therefore, it will be interesting to see the effect it has on the ECR of CO_2_ to CO.

### 4.3 Doping of metal electrocatalysts

In this review, we highlighted the benefits of doping in improving the performance of carbon-based electrocatalysts. Doping is also commonly used in other catalyst fields in improving the performance of materials. Interestingly, this is not widely explored in terms of the ECR process. This can be due to the high production cost associated with the doping process, which remains a constraint.

### 4.4 Catalyst stability

The particle size of electrocatalysts has a direct influence on their edge to face ratio. Decreasing the particle size results in an increase in active catalytic sites, which contributes to good COOH stability and translates to high durability of high energy efficiency.

Finally, this review presented the electrocatalysts that have the greatest potential in improving if not eliminating the CO_2_ problem plaguing the world today. Despite the numerous challenges, it is believed that focus on the development of these electrocatalysts is crucial for their implementation as a practical solution to reduce CO_2_ in the near future.

## Funding sources

This work is supported by the Singapore Ministry of Education [MOE2017-T2-2-067].

## Author contributions

The manuscript was written through contributions of all authors. All authors have given approval to the final version of the manuscript.

## Conflicts of interest

The authors declare no competing financial interest.

## Supplementary Material
